# *Chnoospora minima* Polysaccharide-Mediated Green Synthesis of Silver Nanoparticles: Potent Anticancer and Antimicrobial Activities

**DOI:** 10.3390/biology14070904

**Published:** 2025-07-21

**Authors:** Lakshika Keerthirathna, Sachini Sigera, Milan Rathnayake, Arunoda Senarathne, Hiruni Udeshika, Chamali Kodikara, Narayana M. Sirimuthu, Kalpa W. Samarakoon, Mohamad Boudjelal, Rizwan Ali, Dinithi C. Peiris

**Affiliations:** 1 Department of Zoology, Faculty of Applied Sciences, University of Sri Jayewardenepura, Nugegoda 10250, Sri Lanka; rangee9183@gmail.com (L.K.);; 2 Genetics & Molecular Biology Unit, Faculty of Applied Sciences, University of Sri Jayewardenepura, Nugegoda 10250, Sri Lanka; kodikarachamali@gmail.com; 3 Department of Chemistry, Faculty of Science, University of Colombo, Colombo 00300, Sri Lanka; milanrathnayake511@gmail.com; 4 Department of Chemistry, Faculty of Applied Sciences, University of Sri Jayewardenepura, Nugegoda 10250, Sri Lanka; 5 Institute for Combinatorial Advanced Research & Education (KDU-CARE), General Sir John Kotelawala Defense University, Ratmalana 10390, Sri Lanka; kalpa.samarakoon@gmail.com; 6 King Abdullah International Medical Research Center (KAIMRC), Medical Research Core Facility and Platforms (MRCFP), King Saud bin Abdulaziz University for Health Sciences (KSAU-HS), Ministry of National Guard Health Affairs (MNGHA), Riyadh 11481, Saudi Arabia; boudjelalmo@mngha.med.sa (M.B.); aliri@kaimrc.edu.sa (R.A.)

**Keywords:** *Chnoospora minima*, brown alga, polysaccharide, silver nanoparticles, green synthesis, anti-cancer, lung cancer cells, antimicrobial, *S. aureus*

## Abstract

Marine algae offer environmentally friendly platforms for green nanoparticle synthesis. This study reports the biosynthesis of silver nanoparticles using polysaccharides isolated from the brown alga *Chnoospora minima* (PAgNPs) and evaluates their therapeutic potential. Fourier Transform Infrared Spectroscopy (FTIR) confirmed algal polysaccharide functional groups. Dynamic Light Scattering (DLS), Scanning Electron Microscopy (SEM), and Energy Dispersive X-ray (EDX) analysis characterized the nanoparticles as spherical (~84 nm average size), stable (zeta potential −18.5 mV), and containing elemental silver without nitrogen. The PAgNPs exhibited potent antioxidant activity (~100% DPPH scavenging) and significant antimicrobial efficacy, particularly against *Staphylococcus aureus* and *Candida* species. Crucially, PAgNPs displayed potent antiproliferative activity against human lung cancer cells (A549, IC_50_: 13.59 µg/mL). In contrast, toxicity to normal Vero cells was significantly lower (IC_50_: 300.2 µg/mL), demonstrating notable cancer cell selectivity (SI 22.1). Moderate activity was observed against MCF-7 breast cancer cells (IC_50_: 100.7 µg/mL). These results demonstrate that *C. minima* polysaccharide facilitates the synthesis of biocompatible AgNPs with promising antimicrobial and selective anticancer capabilities, highlighting their potential for further development as nanotherapeutics.

## Simple Summary

Marine algae are a rich source of natural compounds that can be used in environmentally friendly ways to create nanoparticles. In this study, we used polysaccharides (a type of sugar) extracted from the brown seaweed *Chnoospora minima* to prepare silver nanoparticles (PAgNPs). We found that these PAgNPs were spherical, about 84 nanometers in size, and stable. These PAgNPs showed a strong ability to neutralize harmful free radicals (antioxidant activity) and effectively stopped the growth of several disease-causing microbes, especially *Staphylococcus aureus* and *Candida* species. Importantly, the PAgNPs were very effective at killing human lung cancer cells (A549; IC_50_: 13.59 µg/mL) while being significantly lower in toxicity towards normal Vero cells (IC_50_: 300 µg/mL), resulting in excellent selectivity (SI = 221). They also showed moderate activity against breast cancer cells (MCF-7). Our findings suggest that polysaccharides from *C. minima* can be used to produce biocompatible silver nanoparticles with promising dual antimicrobial and selective anticancer properties, making them interesting candidates for new nanomedicines.

## 1. Introduction

Nanotechnology, in conjunction with nanomedicine, has added new insight into the therapeutic and pharmacological arenas. Nanotechnology is a dynamic field comprising chemistry, physics, biology, and engineering, which has a high proficiency in disease treatment delivery methods and manufacturing new devices for molecular and cellular biology [1]. Nanoparticles (NPs) are ideal for biological applications in terms of treatment due to their shape, size, and unique optical and thermal properties [2]. The metallic NPs, such as gold, silver, iron, zinc, and metal oxide nanoparticles, showed interest in medical applications [1]. Among metallic nanoparticles, silver nanoparticles (AgNPs) are widely used as an antitumor agent [3]. Similarly, AgNPs synthesized using chemical reduction, such as photochemical and electrochemical techniques, use hazardous reducing agents. The synthesis process itself is costly and poses risks to the environment [4]. However, at higher concentrations, AgNPs tend to be toxic [5]. Metallic nanoparticles are mainly produced using chemical procedures such as photochemical and electrochemical methods and radiation [6]. Owing to the harmful nature and the cost of these metallic nanoparticles [4], the focus is shifting toward “green synthesis” of nanoparticles using fungi, bacteria, plants, and algae [7]. The NPs synthesized using green synthesis are highly stable, cost-effective, nontoxic, and eco-friendly [8]. The green synthesis of characteristically safer AgNPs depends on adopting the basic requirements of green chemistry, including a solvent medium, a reducing agent, and a non-hazardous stabilizing agent [9]. NP synthesis using microorganisms, enzymes, plants, and algae has been proposed as a feasible, environmentally friendly alternative to chemical and physical techniques. Further, the ability of algae to accumulate metals and reduce metal ions makes them a superior contender for the biosynthesis of NPs, and therefore, they are ideal candidates for the synthesis of metallic NPs [10]. Also, polysaccharides and phenolic compounds act as a capping agent and thus improve their stability. Currently, the interest in marine macroalgae has increased due to identifying potent natural compounds synthesized in them as secondary metabolites [11]. The biological properties of these secondary metabolites have been studied thoroughly, and they are known to exhibit anticancer, anti-inflammatory, antiviral, and antimicrobial activities [11].

**Figure 1:**
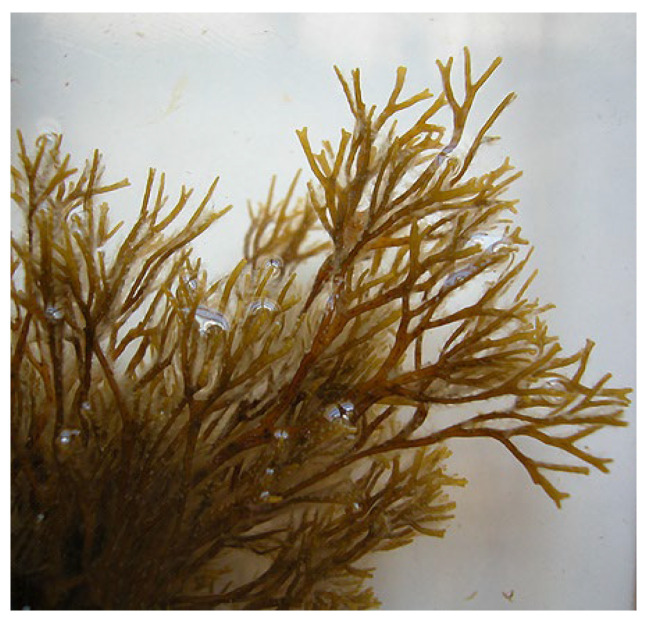
Macroscopic morphology of brown seaweed *Chnoospora minima*.

Cancer is the primary cause of death worldwide, with 9.6 million deaths and 18.1 million cases in 2018 [12,13]. It is caused by the unrestrained proliferation of cells followed by a subsequent invasion of healthy cells and tissues [14,15]. Among cancers, breast cancer is the most frequent among women in developed and developing countries, affecting 1 in 8 of the female population [16]. Chemotherapy and radiotherapy are the first lines of treatment currently used against cancer, often accompanied by severe side effects that compromise patients’ quality of life. Most chemotherapeutic drugs are cytotoxic and immunotoxic [17]. Therefore, contrary to the established treatment methods for cancer, it has become a vital goal for researchers to identify and develop new, effective therapeutic agents. Hence, the efficient carrier properties of nanoparticles can be employed to treat cancer by either passive or active processes. NPs can surpass a few of the hurdles in conventional cancer treatments. They increase the drug’s efficacy due to its site specificity and targeted activity, as NPs evade the immune responses and cross the impermeable cell membranes [18]. Also, the leaky vasculature nature of the cancerous tissue allows NPs to diffuse quickly into the cancerous cells and induce cell suicide [19]. Polysaccharides from algae can synthesize highly stable nanoparticles to overcome the toxicity of AgNPs. *Chnoospora minima*, (Figure 1) a marine brown alga (family: Scytosiphonaceae), was selected for this study due to its high polysaccharides with reported biological activities and reported antimicrobial activities, including antioxidant properties [19]. Previously, it was recorded that the polysaccharide exhibits potent anti-inflammatory activity [19] and antioxidant activity [20]. The major polysaccharides found in brown algae are fucoidan and alginic acid [21]. Owing to the significance of algal polysaccharides, we tested antiproliferative activities using three different cell lines. Given the significance of nanoparticles in cancer therapy, we report for the first time the biosynthesis of silver nanoparticles (AgNPs) using natural polysaccharides derived from *C. minima.* Further, we validated the anticancer activity of these algal polysaccharide-based AgNPs (PAgNPs) against breast carcinoma (MCF-7) cells and other cancer cell lines, along with their antimicrobial and antioxidant potential.

## 2. Materials and Methods

### 2.1. Algal Material and Polysaccharide Extraction

The brown marine macroalgae *Chnoospora minima* were collected from Mirissa, Coral Reef (5°56′38.66″ N, 80°27′19.4″ E) in the Southern Province of Sri Lanka from January to September 2024. The permission to collect was obtained from the Department of Wildlife, Sri Lanka (permit no. WL 3/2/7/2023). The collected seaweed was placed into sterile polyethylene bags and transferred to the laboratory under controlled conditions for extraction procedures. The algae were authenticated by Dr. Kalpa Samarakoon, Kothalawala Defense University, Sri Lanka. The collected algae were washed in seawater to remove the macroscopic epiphytes and other extraneous matter and rinsed in distilled water. The specimen was shade-dried and coarsely powdered. Briefly, 300 g of the dried seaweed powder was depigmented with acetone, followed by hot water extraction at 90–95°C for 3–4 h. The extract was then filtered through Whatman No. 3 filter paper, concentrated to 1/4th of the original volume, cooled, and precipitated with three volumes of 95% ethanol overnight at 4 °C. The precipitate was collected by centrifugation at 1200 rpm at 4°C and dried at 40 °C to obtain the brown sulphated polysaccharide [22].

### 2.2. Green Synthesis of Silver Nanoparticles (PAgNPs)

Thirty milligrams of a crude polysaccharide extract of the algae were dissolved in 90 mL of sterile distilled water with continuous stirring at 70 °C. Subsequently, 1 mL of a 0.1 mM AgNO_3_ solution was added to the obtained solution dropwise with constant stirring, and the solution pH was adjusted to 10. The final volume was completed to 100 mL with sterile distilled water and kept in a magnetic stirrer at 70 °C under constant stirring for 20 min. The reduction of silver ions to AgNPs was routinely monitored by visual inspection of the solution and UV–vis spectra (Shimadzu UV-1900i, Tokyo, Japan) [23].

### 2.3. Characterization of Polysaccharide and PAgNPs

#### 2.3.1. Fourier Transform Infrared (FTIR) Spectroscopy

Commercially available standards of polysaccharides, alginate (alginic sodium salt, D-7924), and fucoidan were purchased, and alginic acid was used as a standard. At room temperature, sodium alginate (50 mg) was dissolved in 0.08 M of phosphate buffer solution. Then, 18M H_2_SO_4_ was added, making the final acid concentration 1M. The solution was left for 30 min to allow the sample to precipitate. Upon centrifuging, the residue was resuspended and used as a standard of alginic acid for FTIR spectroscopy (Thermo Scientific Nicolet S10 FTIR Spectrometer, Fishers, Indianapolis, IN, USA). The FTIR spectra of algal polysaccharide standards were recorded using a Nicolet S10 FTIR spectrometer with the KBr pellet method [24].

#### 2.3.2. Biochemical Characterization of Seaweed Extract

The phenol-sulfuric method was employed to determine the total carbohydrate content with minor modifications [25]. In a hot, acidic medium, carbohydrates were dehydrated to hydroxymethylfurfural, forming a green-coloured product with phenol, which has an absorption maximum of 490 nm. The total monosugar content was measured using the dinitro acid (DNS) method [26]. Further, the total protein content was analyzed using a BCA protein assay kit, while the sulphate content was measured using the BaCl_2_ gelation method [27].

#### 2.3.3. UV–Visible (UV-Vis) Spectroscopy

The colour change in the bioreduction reaction medium was monitored by periodic sampling of the reaction solution and by measuring using UV–Vis spectroscopy. The UV–Vis spectra of the AgNPs were recorded as a wavelength function using a UV–Vis spectrophotometer (Shimadzu UV-1900i, Shimadzu, Tokyo, Japan), which facilitated the identification of nanoparticle formation (UV–Vis). The absorbance of the reaction mixture was measured in the 200–800 nm range. Silver nitrate was used for the baseline correction.

#### 2.3.4. Dynamic Light Scattering (DLS) and Zeta Potential

The DLS measures the average particle size and size distribution profile. Upon sonicating the nanoparticles dispersed using distilled water, the polydispersity index (PDI) and zeta potential of the synthesized PAgNPs were taken at room temperature using a Malvern Zetasizer Nano ZS Instrument (Malvern, Worcestershire, UK). Each sample was measured three times for accuracy.

#### 2.3.5. Scanning Electron Microscopy (SEM) and Energy Dispersive X-Ray (EDX) Analysis

The morphology and element composition of PAgNPs were analyzed using SEM and EDX techniques. A drop of the PAgNP suspension was placed on a carbon-coated copper grid, air-dried, and then coated with a thin layer of carbon. Images were obtained using an SEM (Hitachi SU6600X-SEM, Hitachi, Tokyo, Japan) equipped with an electrical beam energy of 10 keV. EDX analysis was carried out by viewing the samples in a scanning electron microscope (FEI, INSPECT S50 with Everhart-Thornley detector, Hillsboro, OR, USA) operating at 10 kV. Droplets of the AgNP sample were placed separately on aluminium stubs without metallization and left in the dark for three days in closed containers until completely dry for subsequent analysis in the equipment. Analysis was conducted using the EDX with Aztec (Morristown, NJ, USA), MountainsMap version 8.2.

#### 2.3.6. Raman Spectroscopy

Raman spectroscopy is a powerful optical technique based on the inelastic scattering of light and, together with IR, is one of two complementary vibrational spectroscopy methods for molecular and material characterization [28]. Hence, to identify the molecules absorbed on the surface of the synthesized PAgNPS, Raman spectra were recorded using a Raman spectrometer (WITec Instrument Corp., Concord, MA, USA) upon amplifying the Raman signals using the electromagnetic field generated by the excitation of localized plasmon resonance (LSPR).

#### 2.3.7. Total Phenolic Content (TPC)

The TPC of the algal polysaccharide extract and the PAgNPs was determined using the Folin–Ciocalteu method [29] with gallic acid as a standard. Briefly, 500 µL of different water extract concentrations was mixed with 2.5 mL of Folin–Ciocalteu reagent. After 5 min, 2 mL of a Na_2_CO_3_ solution was added, and after 120 min of standing in the dark, the optical density was measured at 760 nm against a blank. The TPC was calculated using a calibration curve of gallic acid and expressed as gallic acid equivalent (GAE) in mg/g of the sample.

### 2.4. Antioxidant Activity (DPPH Radical Scavenging Assay)

The antioxidant activity was evaluated using the 2,2-diphenyl-1-picrylhydrazyl (DPPH) radical scavenging assay [30]. Different concentrations of PAgNPs, *C. minima* polysaccharide (7.813 to 0.061 µg/mL), and a standard antioxidant (ascorbic acid) were mixed with a methanolic solution of DPPH (1 mM in methanol). The mixture was incubated in the dark at room temperature for 30 min. The absorbance was measured at a 517 nm wavelength using a microplate reader. The percentage of DPPH radical scavenging activity (%RSA) was calculated using the formula %RSA = (A_control_ − A_sample_)/A_control_ × 100, where A_control_ is the absorbance of the DPPH solution without the sample, and A _sample_ is the absorbance of the DPPH solution with the sample.

### 2.5. Antimicrobial Susceptibility Testing

#### 2.5.1. Microbial Strains and Culture Conditions

Seven clinically pathogenic organisms were used. Gram-negative: *Escherichia coli* (ATCC 25922), *Pseudomonas aeruginosa* (ATCC 25853), and *Klebsiella pneumoniae* (ATCC 13883); Gram-positive: *Staphylococcus aureus* (ATCC 25923) and *Bacillus cereus* (ATCC 11778); and fungal strains: *Candida albicans* (ATCC 10231) and *Candida glabrata* (ATCC 90030). The pathogenic organisms were obtained from the Faculty of Medicine Sciences, University of Sri Jayewardenepura, Sri Lanka. Bacterial strains were cultured on Muller Hinton Agar at 37 °C and fungal strains were cultured on Dextrose Agar/Broth at 28 °C.

#### 2.5.2. Agar Well Diffusion Assay

Antimicrobial activity was assessed using the agar well diffusion method. Microbial cultures adjusted to 0.5 McFarland standard were spread onto agar plates. Sterile filter paper discs (diameter: 6 mm) were impregnated with PAgNP suspension (5 × 10^8^ CFU/mL) and incubated for 24 h at 37. A disc impregnated with 10 µg gentamicin was used as the positive control for bacteria, and a 25 µg fluconazole disc was used for fungal strains [31]. The agar plates were incubated for 24 h at 37 °C. The antimicrobial activities were evaluated by measuring the diameter of the inhibition zones at 500 ppm concentrations in millimetres (mm). Experiments were performed in triplicate.

The McFarland standard is a turbidity measurement used to estimate the concentration of microbial cells in a suspension. A 0.5 McFarland standard corresponds to approximately 1.5 × 10^8^ colony-forming units (CFUs)/mL, ensuring uniform inoculum density across antimicrobial susceptibility tests. It is typically prepared by mixing barium chloride and sulfuric acid to create a reproducible level of turbidity, which is then matched visually or measured spectrophotometrically.

### 2.6. Cell Lines and Cell Culture

Human lung carcinoma (A549; ATCC No: CRM-CCL-185), human acute promyelocytic leukemia cell line (HL60; ATCC No: CCL-240), and human breast cancer cell line (MCF7; ATCC No: HTB-22) were used to study the antiproliferative activity of PAgNPs. The Vero cell line (ATCC-CCL 81) was employed to study the toxicity to normal cells. Cancer cells were grown in Dulbecco’s Modified Eagle’s Medium (DMEM) supplemented with 10% FBS, 50 units/mL of penicillin, 50 ug/mL of streptomycin (GIBCO), and [32]. The cells were cultured at a density of 1 × 10^6^ cells/mL at 37 °C in a humidified 5% CO_2_ atmosphere. The cells were sub-cultured when 80–90% confluency was reached. Upon treatment with the polysaccharide AgNPs or the standard drug, the treatment, the cells were observed after 24 h of treatment.

The majority of cell line work was carried out in Saudi Arabia. However, in Sri Lanka, we conducted cell cytotoxicity activity for the available cell line of MCF7 for both crude polysaccharide extract and green-synthesized silver nanoparticles using the standard MTT assay upon 24h treatment.

### 2.7. Antiproliferative Activity (CellTiter-Glo Assay)

Cell viability was assessed using a CellTiter-Glo assay. Cells were seeded at a predetermined density on a white 96-well plate, treated with polysaccharide extract or mitoxantrone (standard drug) at different concentrations, and incubated for 24 h. Cell lysis and viability assessment were performed using the CellTiter-Glo^®^ Luminescent Cell Viability Assay (Promega, Durham, NC, USA) following the manufacturer’s protocol. Upon incubation for 15 min in the dark, the luminescence was read using the Envision plate reader (Perkin Elmer, Waltham, MA, USA). The percentage of cell growth was calculated relative to the DMSO (dimethyl sulfoxide)-treated cells [33] (DMSO was used as a vehicle control as it served as the solvent for dissolving the polysaccharide extract and mitoxantrone, allowing assessment of their effects independent of any influence from the solvent itself). For the green-synthesized AgNPs and the standard drug, the concentrations ranging from 50 to 1800 and 0.01 to 100 µg/mL were tested, respectively. The percentage of cell viability was calculated as % Viability = (Ab_treated_/Abs_control_) × 100. The IC_50_, or concentration inhibiting 50% of cell growth, values were determined from dose–response curves. The selectivity index (SI) for A549 cells was calculated as SI = IC_50_ of Vero cells/IC_50_ of cancerous cells.

### 2.8. Statistical Analysis

All experiments were performed in triplicate, and data are presented as mean ± standard deviation (SD). Statistical analysis was performed using GraphPad Prism version 9. Differences between groups were analyzed using ANOVA followed by the Tukey test. A *p*-value < 0.05 was considered statistically significant.

## 3. Results

### 3.1. Characterization of Algal Polysaccharide

**Figure 2:**
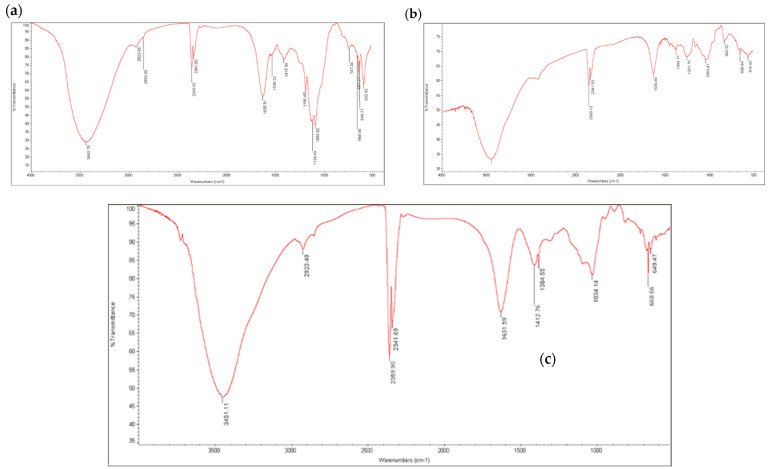
FT-IR spectrum of polysaccharide extracted from *C. minima*. (**a**) Spectrum of the extracted polysaccharide; (**b**) standard fucoidan spectrum; (**c**) standard alginic acid spectrum.

The FTIR spectrum of the polysaccharide extracted from *C. minima* (Figure 2a) was compared with standard fucoidan (Figure 2b) and alginic acid (Figure 2c). Common to all spectra, broad bands centred at 3400–3500 cm^−1^ (O-H stretching), which was typical of all polysaccharides, and weak signals at ~2926 cm^−1^ (C-H stretching) were observed, indicating sugar rings. A strong peak at 1614 cm^−1^ in the *C. minima* polysaccharide spectrum was attributed to the asymmetric stretching vibration of carboxylate anions (COO), indicating the presence of uronic acids, which are characteristic components of brown algae polysaccharides or the alginates. Peaks in the 1210–1260 cm^−1^ range, associated with asymmetric S=O stretching of sulphate esters (O-SO_3_), were also noted. The fingerprint region (950–750 cm^−1^) showed typical polysaccharide absorptions (Figure 2a).

The total carbohydrate content of the extracted polysaccharide, using glucose as the standard, was determined to be 20 ± 0.417%. The protein and sulphate content were measured as 3.01 ± 0.4 and 11.2 ± 0.65, respectively. The total carbohydrate, sulphate, and protein content accounted for approximately 32%, and hence, the remaining mass of the dried extract likely comprises phenolic compounds (see TPC analysis in Section 3.5).

### 3.2. Synthesis and Visual Observation of PAgNPs

**Figure 3:**
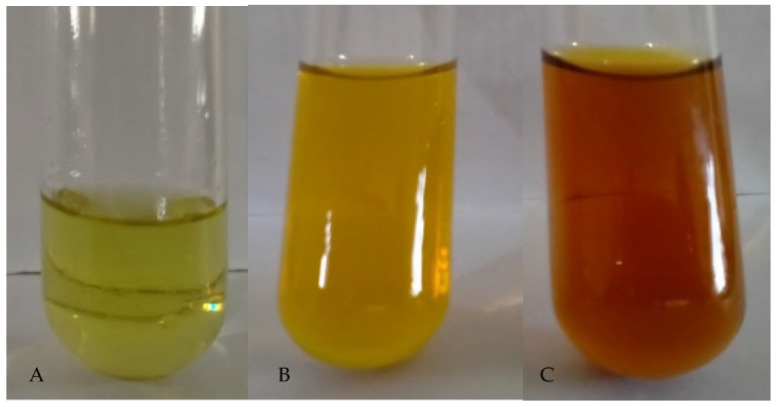
Visual observation of the colour changes in the reaction mixture before and after adding AgNO_3_ to crude polysaccharide isolated from *C. minima*. (**A**) crude polysaccharide; (**B**) after 30 min; (**C**) after 60 min.

The addition of the *C. minima* polysaccharide extract to an aqueous AgNO_3_ solution resulted in a colour change to yellowish-brown, indicating the formation of PAgNPs (Figure 3). This colour change is attributed to the Surface Plasmon Resonance (SPR) phenomenon, where, when the light hits the AgNPs, the conduction electrons oscillate collectively against the metal lattice, and these oscillations absorb and re-emit light at a specific wavelength, giving rise to vivid colours seen in the suspension [33]. For AgNPs around 10–100 nm, SPR absorption usually peaks at ~400–450 nm, which corresponds visually to yellowish-brown hues [34].

### 3.3. Spectroscopic Characterization of PAgNPs

**Figure 4:**
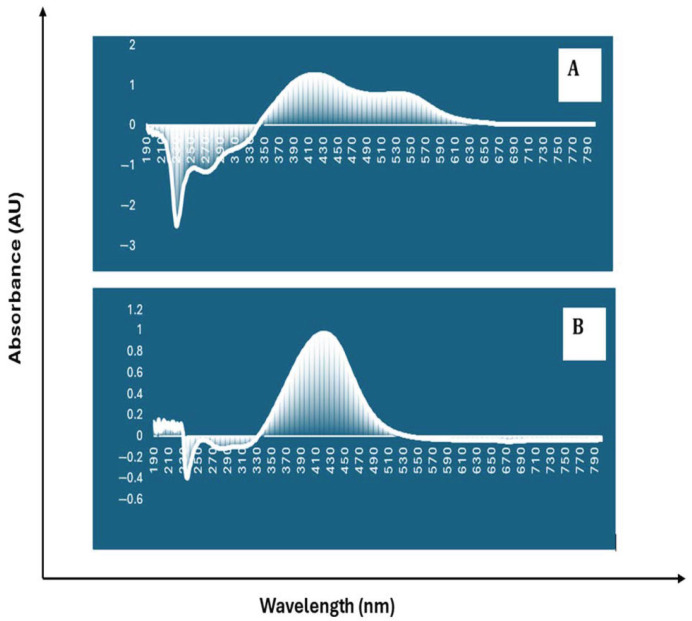
UV–vis spectrum analysis of biosynthesized nanoparticles capped by polysaccharide of *C. minima.* (**A**) Absorbance after 30 min and (**B**) absorbance after 60 min.

UV-Vis spectroscopic analysis confirmed the formation of PAgNPs. At 30 min, the absorbance peak was not intense (Figure 4A). However, after 60 min, the intensity of the absorbance peak increased significantly, with a characteristic SPR band centred at 420 nm (Figure 4B), confirming PAgNP synthesis.

### 3.4. DLS, SEM, and EDX Analyses of PAgNPs

**Figure 5:**
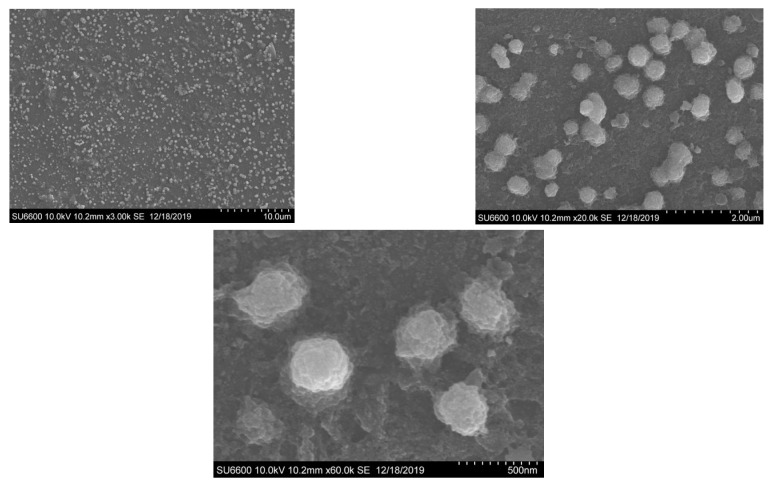
SEM images of green-synthesized Ag-NPs capped with polysaccharides isolated from *C. minima*.

**Figure 6:**
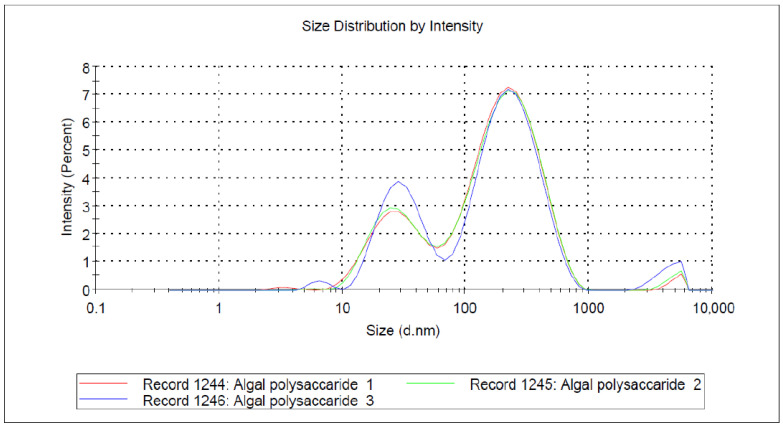
DLS image showing the size distribution of the green-synthesized Ag-NPs. Different coloured lines represent the *n* = 3 runs of the samples.

**Figure 7:**
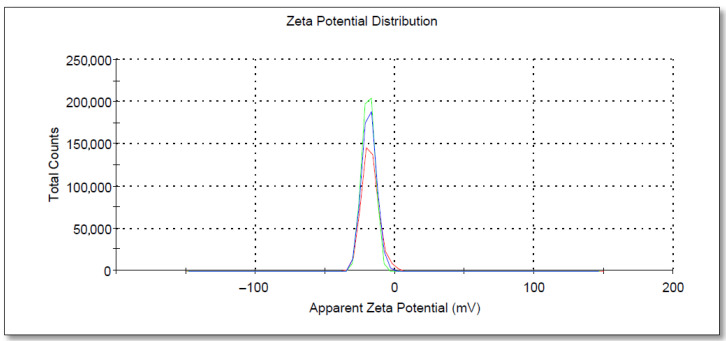
Zeta potential reading of green-synthesized Ag-NPs.

SEM analysis showed that the PAgNPs were roughly spherical with low aggregation, and sizes ranged from 50 to 300 nm (Figure 5). DLS analysis indicated an average hydrodynamic size of 84 nm for the PAgNPs (Figure 6) with a PDI value of 0.5, which indicates a polydisperse population of nanoparticles rather than a monodispersed population, which would usually give a PDI of less than 0.2. The polydispersity of the nanoparticle population is also shown in the DLS graphs, where three peaks are visible: a 74% intense peak 1 around 250 nm, a 24% intense peak 2 around 30 nm, and a 1.2% intense peak 3 around 5000 nm. The zeta potential of the PAgNP suspension was measured at −18.5 mV (Figure 7).

**Figure 8:**
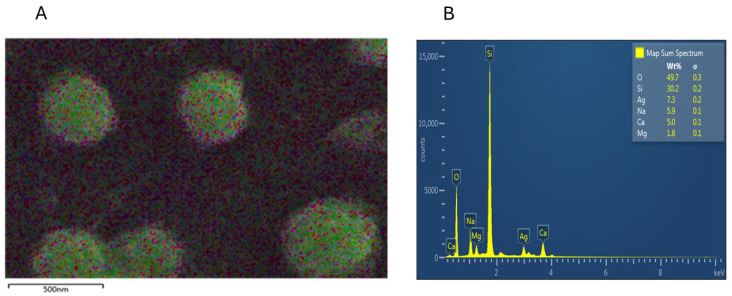
EDX patterns of synthesized Ag-NPs. (**A**) EDX layered image and (**B**) EDX spectra recorded from film of green-synthesized Ag-NPs with different X-ray emission labels.

EDX analysis confirmed the elemental composition of the PAgNPs. A strong elemental silver signal was observed (Figure 8A). The quantitative analysis revealed the wt.% of silver (Ag) at 7.3 wt.%. Other prominent elements detected included oxygen (O) at 49.7 wt.%, sodium (Na) at 5.9 wt.%, 30.2% silicon (Si) at %wt., calcium (Ca) at 5.0 wt.%, and magnesium (Mg) at 1.8 wt.% (Figure 8B). EDX analysis confirmed the successful synthesis of polysaccharide-mediated silver nanoparticles (PAgNPs) by identifying the elemental composition of the sample. A strong signal for silver (Ag) at 7.3 wt.% indicated the presence of elemental silver and supported the formation of AgNPs. The high oxygen (O) content (49.7 wt.%) is attributed to the oxygen-rich functional groups (such as hydroxyl, carboxyl, and sulphate) present in the polysaccharide capping agents, as well as the possible formation of a thin silver oxide (Ag_2_O) layer on the nanoparticle surface during synthesis or storage. A notable signal for silicon (Si) at 30.2% wt. is from the silicon-containing substrate, the glass slide used for SEM-EDX sample preparation. The electron beam during EDX analysis can penetrate or interact with the underlying substrate, thus contributing to the Si signal.

Other elements, including sodium (Na), calcium (Ca), and magnesium (Mg), are commonly found in marine polysaccharides derived from seaweeds and reflect the mineral content associated with the natural polysaccharide matrix or residual salts from the extraction process.

### 3.5. Raman Spectroscopy and Total Phenolic Content (TPC) of PAgNPs

**Figure 9:**
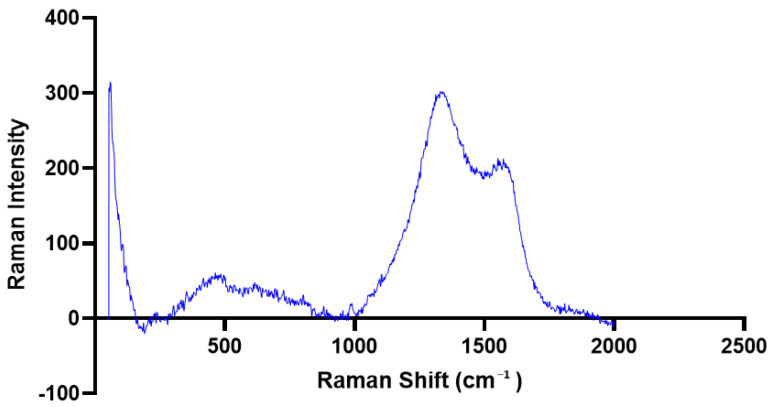
Raman spectrum of green-synthesized polysaccharide-based Ag-NPs.

**Figure 10:**
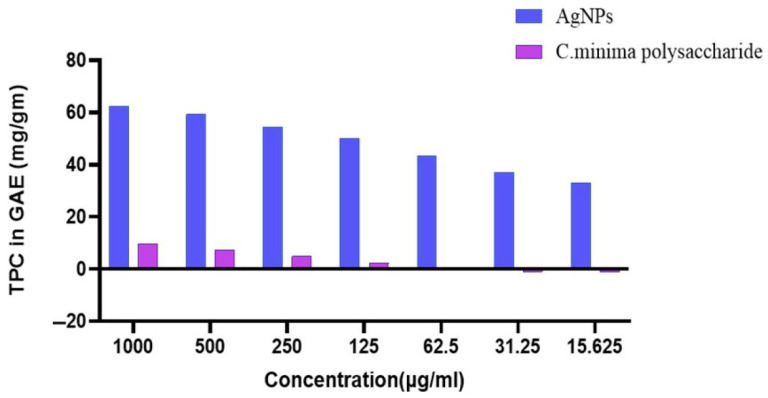
Total phenol content in gallic acid equivalent (µg/gm) in polysaccharide extract (pink colour) and biosynthesized Ag-NPs capped with *C. minima* polysaccharide (blue colour).

The Raman spectrum of the synthesized PAgNPs (Figure 9) showed peaks in the 500–1600 cm^−1^ region (aromatic C-C stretching) and the 1300–1370 cm^−1^ region (NO_2_ stretching). A notable peak at 1500–1600 cm^−1^ was attributed to aromatic C-C stretching. The PAgNPs exhibited significantly higher TPC compared to the algal polysaccharide extract alone across all tested concentrations (Figure 10).

### 3.6. Antioxidant Activity of PAgNPs

**Figure 11:**
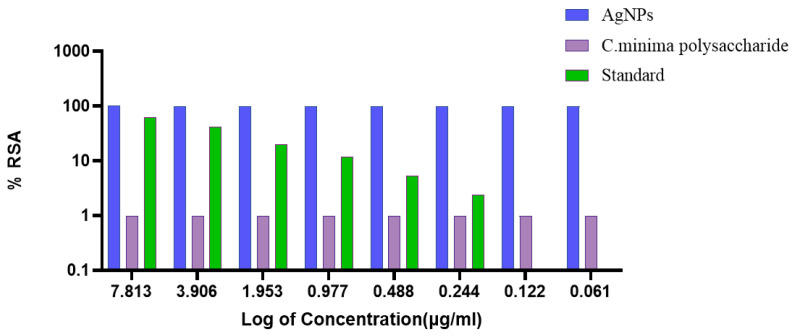
Percentage of radical scavenging DPPH activity with different concentrations of biosynthesized AgNPs capped with polysaccharides isolated from *C. minima* (blue colour), polysaccharides of *C. minima* (pink colour), and the standard (green colour). Standard: ascorbic acid.

The DPPH radical scavenging activity (%RSA) of PAgNPs, *C. minima* polysaccharide, and a standard was evaluated (Figure 11). The PAgNPs demonstrated the highest %RSA (nearly 100%) across all tested concentrations (7.813 to 0.061 µg/mL), showing superior antioxidant potential compared to the polysaccharide and the standard in this assay format (IC_50_ < 0.061 µg/mL). The IC_50_ values obtained for C. minima polysaccharide and the standard ascorbic acid were 7.813 and 4.5 µg/mL, respectively. The results highlighted that the PAgNPs are highly effective antioxidants, although the concentrations used were too high to allow for the determination of the IC_50_ value, as the response was saturated even at the lowest concentration tested. The *C. minima* polysaccharide consistently showed the lowest %RSA. No clear dose-dependent effect was observed for any of the substances within the tested range.

### 3.7. Antimicrobial Susceptibility of PAgNPs

**Figure 12:**
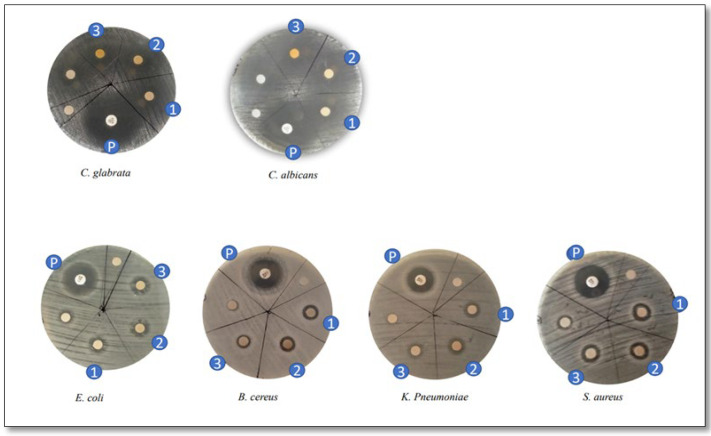
Inhibition zones of microbial strains used (P: positive control, while 1, 2, and 3 represent *n* = 3 technical replicates: treatment with *C. minima* PAgNO_s_). Unsigned disc refers to negative control.

PAgNPs exhibited antimicrobial activity against all seven tested clinical pathogenic organisms, with the 500 ppm (0.5 mg/mL) concentration producing diffuse inhibition zones (Figure 12; Table 1). Against fungal strains, PAgNPs showed a higher inhibitory effect against *C. albicans* (inhibition zone: 15 ± 0.02 mm) compared to its effect on *C. glabrata* (12 ± 0.1 mm). Among bacterial strains, the highest MIC value against Gram-positive bacteria was for *S. aureus* (12 ± 0.015 mm), which was more susceptible than *B. cereus* (8 ± 0.000 mm). *K. pneumoniae* showed an inhibition zone of 8 mm (Table 1).

### 3.8. Antiproliferative Activity of PAgNPs

**Figure 13:**
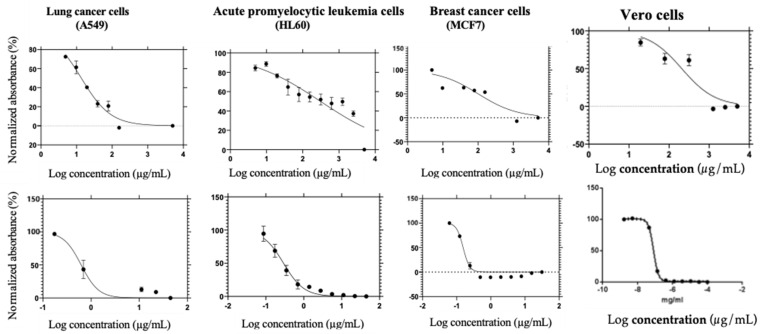
The 50% cell-killing activity (IC_50_) of the human cancer cell lines, including lung cancer (A549), acute promyelocytic leukemia (HL60), breast cancer cells (MCF7), and Vero cells. The top row shows the IC_50_ of the different concentrations of the biosynthesized AgNPs capped with polysaccharides isolated from *C. minima,* while the bottom row is the positive control (Mitoxantrone).

The green-synthesized PAgNPs inhibited the proliferation of A549 (lung cancer cells), HL60 (acute promyelocytic leukemia cells), and MCF-7 (breast cancer cells) in a concentration-dependent manner (Table 2; Figure 13). The IC_50_ values were 13.59 µg/mL for A549 cells, 306.1 µg/mL for HL60 cells, and 100.7 µg/mL for MCF-7 cells. Mitoxantrone, the standard drug, exhibited IC_50_ values of 0.6217 µg/mL (A549), 0.4887 µg/mL (HL60), and 0.2913 µg/mL (MCF-7). The IC_50_ values were 13.59 µg/mL for A549 cells, indicating potent activity. However, considerably higher IC_50_ values were observed for HL60 cells (306.1 µg/mL) and MCF-7 cells (100.7 µg/mL), suggesting significantly lower sensitivity of these cell lines to the PAgNPs compared to A549 cells. Against normal Vero cells, PAgNPs exhibited an IC50 value of 300.2 µg/mL, suggesting an IC_50_ significantly higher than for A549 cells and indicating a favourable selectivity index (SI > 1) for the lung cancer cell line. High values obtained for Vero cells emphasize the non-toxic nature of PAgNPs.

Then, the cytotoxicity of green-synthesized Ag-NPs and the crude polysaccharide was compared using the standard MTT assay against MCF-7 breast cancer cells at 48 h post-incubation. The percent of viable cells (%) treated with various concentrations (ranging from 1.125 to 9.00 μg/mL) of green-synthesized Ag-NPs is shown in Table 3. The green-synthesized Ag-NPs exhibited cytotoxicity against MCF-7 cancer cells in a dose-dependent manner, with the ascending order of percentage cell viability decreasing in the order of 0 < 1.125 < 2.25 < 4.5 < 9 μg/mL, with a 50% inhibitory concentration (IC_50_) of 3.921 μg/mL. According to Table 3, green-synthesized Ag-NPs exhibited potent cytotoxic activity (IC_50_: 3.921 μg/mL) against the MCF-7 cells compared to the crude polysaccharide (IC_50_: > 200 μg/mL).

## 4. Discussion

The green synthesis of nanoparticles using biological entities like marine algae is a promising eco-friendly alternative to conventional chemical methods [7,8]. In this study, polysaccharides extracted from the brown alga *C. minima* were successfully utilized for the synthesis of silver nanoparticles (PAgNPs). The FTIR analysis of the extracted polysaccharide (Figure 2a) indicated the presence of key functional groups characteristic of brown algal polysaccharides such as alginates (strong COO peak at 1614 cm^−1^) and fucoidans (S=O stretching at 1210–1260 cm^−1^) [21]. However, a more detailed structure elucidation study, including precise monosaccharide composition and molecular weight distribution, was beyond the scope of the current work. Nevertheless, the presence of abundant hydroxyls and carboxylates, confirmed by FTIR analysis, provides a strong basis for their use as reducing Ag^+^ ions and capping agents for the formed PAgNPs, contributing to their stability [23]. Further, the presence of 11.2 ± 0.65 sulphate indicates the presence of sulphated polysaccharides. The total carbohydrate content of 20 ± 0.417% further supports the polysaccharide nature of the extract. Nevertheless, the relatively low total carbohydrate content (approximately 20%) in the dried crude extract suggests that other co-extracted molecules also significantly contribute to its mass. Though we did not quantify the TPC, previously, we reported that *C. minima* methanol extract is rich in phenolic, flavonoids, and tannin [11] and it is possible that phytochemical compounds could be major co-extractives. Furthermore, pigments like fucoxanthin and salts may also participate in the reduction of silver ions and thus the stabilization of the PAgNPs. This complex composition of the capping layer, derived from the crude algal extract, could contribute to the observed multifaceted biological activities.

The formation of PAgNPs was confirmed by the colour change to yellowish-brown and the characteristic SPR peak at 420 nm in the UV-Vis spectrum (Figure 3 and Figure 4B), consistent with previous reports on AgNP synthesis [34,35]. The single SPR peak suggests the formation of spherical nanoparticles [36], which was corroborated by SEM imaging (Figure 5). DLS analysis revealed an average PAgNP size of ~84 nm and a PDI of 0.5, suggesting that the silver nanoparticles were of broad size distribution. Similar results for PDI were recorded by Gingora and Gingora [37] with *Salvia officinalis*. However, nanoparticles below 1000 nm are considered acceptable carriers [38]. Future optimization of the synthesis parameters could be explored to achieve a narrower size distribution and lower PDI, potentially enhancing their therapeutic efficacy. The negative zeta potential of −18.5 mV (Figure 7) indicates good colloidal stability of the PAgNPs, preventing aggregation due to electrostatic repulsion. Further, negative zeta potential suggests that polysaccharides have a small influence on the colloidal stability of these NPs. The polysaccharide capped the silver particles and acted as a dispersant for them [37]. The EDX analysis confirmed the presence of elemental silver (7.3 wt.%) along with other elements likely originating from the algal extract or residual salts (Figure 8A,B). The detection of significant amounts of oxygen, carbon, sodium, calcium, magnesium, and silicon is consistent with the NPs being capped by the algal polysaccharide extract. Similar elemental profiles have been reported for AgNPs synthesized from other seaweeds [39]. The relatively low weight percentage of silver suggests that a substantial portion of the analyzed material consists of the organic capping layer derived from the crude extract.

Raman spectroscopy (Figure 8) indicated the presence of aromatic C-C stretching (1500–1600 cm^−1^), suggesting that phenolic compounds, possibly phlorotannins, which are common in brown algae [21], are adsorbed on the PAgNP surface. The peak observed at the 1300–1370 cm^−1^ region suggests NO_2_ stretching that originated from nitrate ion residues of AgNO_3_ precursors used in the synthesis. These phenolic compounds, along with polysaccharides, likely contribute to the capping and stabilization of PAgNPs and may also enhance their biological activities [28,40]. This is supported by the significantly higher TPC in PAgNPs compared to the polysaccharide extract alone (Figure 10), suggesting an enrichment or enhanced detectability of phenolics associated with the nanoparticles during the synthesis process [27].

The PAgNPs demonstrated potent antioxidant activity, scavenging nearly 100% of DPPH radicals across the tested concentrations (Figure 11), and it did not reach the maximal activity with the tested range. The polysaccharide extract exhibited lower scavenging ability. However, the standard, ascorbic acid, displayed a dose-dependent increase. Nearly 100% antioxidant activity was obtained, which may be because the PAgNPs had the characteristics of smaller particle size and larger specific surface area than other polysaccharide nanoparticles, which can integrate with free radical scavengers and had better antioxidant activity. Further, the intermediate compounds formed by polysaccharides and metal ions during the formation of silver nanoparticles may also play a role in scavenging free radicals. Finally, the polysaccharide extract is likely due to the synergistic effect of the silver core and the enriched layer of capping agents, including phenolic compounds known for their antioxidant properties [41].

In terms of antimicrobial activity, the PAgNPs were effective against a range of pathogenic bacteria and fungi (Figure 13; Table 1) at 500 ppm. The notable efficacy against *S. aureus* (Gram-positive) and *Candida* species is significant, as these are common human pathogens. The mechanism of AgNP antimicrobial action is multifaceted, involving adherence to and disruption of the cell membrane, inhibition of respiratory enzymes, interference with DNA replication, and generation of reactive oxygen species (ROS) [42]. Though the PAgNPs showed efficacy against Gram-positive bacteria, further studies should be conducted to determine the inhibitory and minimum bacterial concentration to provide a more quantitative measure of the nanoparticle potency against these pathogens.

Crucially, the PAgNPs exhibited potent and selective anticancer activity. The IC_50_ value against A549 lung cancer cells (13.59 µg/mL) was particularly promising and comparable in potency (though numerically higher) to the standard drug mitoxantrone when considering the crude nature of the PAgNPs. Hence, the *Chnoospora minima* polysaccharide-mediated silver nanoparticles displayed potent and selective antiproliferative activity against A549 human lung cancer cells (IC_50_: 13.59 µg/mL). In contrast, their IC_50_ against normal Vero cells was 300.2 µg/mL, resulting in a highly favourable selectivity index (SI = 22.1) for the lung cancer cells over normal Vero cells. Crucially, the PAgNPs exhibited potent and selective anticancer activity, particularly against A549 lung cancer cells (IC_50_: 13.59 µg/mL). This potency was notably higher than that observed against MCF-7 breast cancer cells (IC_50_: 100.7 µg/mL) and HL60 acute promyelocytic leukemia cells (IC50: 306.1 µg/mL). This differential cytotoxicity suggests cell-type-specific mechanisms of action or varying susceptibility to the PAgNPs. This selectivity is a highly desirable trait for anticancer agents [33]. Moderate activity was observed against MCF-7 breast cancer cells, while HL60 cells were less sensitive. Previous studies have shown that AgNPs can induce apoptosis and DNA fragmentation and inhibit cancer cell proliferation [43,44]. The small size (~84 nm) and surface charge of the PAgNPs likely facilitate their penetration into the tumour microenvironment and cellular uptake [45,46]. The polysaccharide capping may further enhance biocompatibility and targeted delivery. The induction of ROS by AgNPs is a known mechanism for DNA damage and apoptosis in cancer cells [47], and the PAgNPs exhibiting a higher selectivity index and less toxicity to DNA-intact Vero cells suggests a cancer-specific mode of action [47]. Further, when we tried to compare the anticancer activity of PAgNPs and the crude polysaccharide extract using the MCF7 cell line, we observed a 50% inhibitory concentration for the crude polysaccharide of 200 μg/mL. Similar results (IC_50_: 200 μg/mL) were obtained by Vaikundamoorthy et al. [48] with polysaccharides isolated from *Sargassum wightii* against MCF-7 breast cancer cells. According to Meena et al. [49], green-synthesized Ag-NPs using the aqueous leaf extract of *Cucumis prophetarum* exhibited anticancer activity against MCF-7 cells with an IC_50_ value of 65.6 μg/mL. Hence, according to the results of antiproliferative activity, the green-synthesized Ag-NPs are more toxic to MCF-7 cells than the crude polysaccharide from *C. minima*. Hence the results showcase the importance of targeted drug delivery using green-synthesized AgNPs.

The study demonstrates that *C. minima* polysaccharides are effective agents for the green synthesis of biocompatible PAgNPs. The presence of both polysaccharides and potentially co-extracted phenolic compounds as capping agents likely contributes to the observed stability and multifaceted biological activities. While the observed bioactivities are attributed to the PAgNPs, it is acknowledged that the polysaccharide capping agent itself might possess some inherent biological effects. Although the polysaccharide extract showed lower antioxidant activity compared to the PAgNPs (Figure 11), its independent anticancer and antimicrobial efficacy was not determined in this study. Therefore, future investigations will critically include the polysaccharide extract alone as a control to precisely delineate its contribution versus that of the silver core or any synergistic effects within the PAgNP formulation. The strong, selective anticancer activity against A549 lung cancer cells, coupled with significant antimicrobial and antioxidant properties, positions these PAgNPs as promising candidates for further nanotherapeutic development. Future studies should also focus on elucidating the precise molecular mechanisms underlying their selective anticancer effects and optimizing their formulation for in vivo applications. 

## 5. Conclusions

In conclusion, this study demonstrates the successful green synthesis of AgNPs (PAgNPs) using a polysaccharide extract from *Chnoospora minima*, resulting in a nanomedicine candidate with significant therapeutic potential. The synthesized PAgNPs were thoroughly characterized, revealing stable, spherical nanostructures (~84 nm). The most significant outcome of this research is the potent and highly selective anticancer activity of the PAgNPs on A549 lung cancer cells (IC_50_: 13.59 µg/mL), coupled with a high selectivity index (SI) of 22.1 over normal Vero cells. This suggests a promising therapeutic window, particularly for lung cancer applications. The potent A549 activity warrants further investigation into the underlying molecular mechanisms. This targeted cytotoxicity is complemented by the PAgNPs’ valuable secondary properties, including potent antioxidant activity and broad-spectrum antimicrobial efficacy against several pathogenic microbes. These multifunctional attributes underscore the potential of using *C. minima* polysaccharides to create biocompatible nanostructures that can combat disease on multiple fronts. Our findings strongly advocate for the further development of these PAgNPs as a novel class of marine-derived nanotherapeutics, with future work focusing on elucidating the molecular mechanisms behind their selective anticancer action.

## Figures and Tables

**Table 1:** Inhibition zones (mm) of microbial strains treated with standard antibiotics or biosynthesized PAgNPs capped with *C. minima* polysaccharides at 500 ppm concentration. Data are mean ± SD (*n* = 3).

Microbial Strains	Inhibition Zone (mm)	
	Fluconazole	Ag-NPs
*C. albicans*	34 ± 0.04	15 ± 0.02
*C. glabrata*	35 ± 0.05	12 ± 0.1
	**Gentamicin**	**Ag-NPs**
*E. coli*	22 ± 0.04	8 ± 0.00
*P. aeruginosa*	20 ± 0.03	8 ± 0.00
*K. Pneumoniae*	15 ± 0.01	8 ± 0.00
*S. aureus*	18 ± 0.02	12 ± 0.015
*B. cereus*	18 ± 0.02	8 ± 0.000

**Table 2:** IC_50_ values of positive control (Mitoxantrone) and AgNPs capped with algal polysaccharide extract of *Chnoospora minima* for different cell lines.

	Mitoxantrone (Standard Drug)	Polysaccharide-AgNPs
Cells	IC_50_ Value (µg/mL)	
A459	0.627	13.59
HL60	0.291	306.1
MCF7	0.153	100.7
Vero	8.54	300.2

**Table 3:** The 50% inhibitory concentration (μg/mL) or IC_50_ of green-synthesized silver nanoparticles and the crude polysaccharide extract of *C. minima* against MCF-7 breast cancer cells.

Extract Type	IC_50_ (μg/mL)
Polysaccharide-based silver nanoparticles	3.921
Crude polysaccharide	200
Vero cell line	0.604

## Data Availability

The original contributions presented in this study are included in the article. Further inquiries can be directed to the corresponding author.

## Abbreviations

The following abbreviations are used in this manuscript:

PAgNPs	Polysaccharide-based Silver Nanoparticles
AgNPs	Silver Nanoparticles
FTIR	Fourier Transform Infrared Spectroscopy
DLS	Dynamic Light Scattering
TPC	Total Phenolic Content
EDX	Energy Disperse X-ray
MIC	Minimum Inhibitory Concentration
